# Curcumin and EGCG combined formulation in nanostructured lipid carriers for anti-aging applications

**DOI:** 10.1016/j.ijpx.2025.100323

**Published:** 2025-02-25

**Authors:** Chidchanok Prathumwon, Songyot Anuchapreeda, Kanokwan Kiattisin, Pawaret Panyajai, Panikchar Wichayapreechar, Young-Joon Surh, Chadarat Ampasavate

**Affiliations:** a Department of Pharmaceutical Sciences, Faculty of Pharmacy, Chiang Mai University, Chiang Mai 50200, Thailand; b Division of Clinical Microscopy, Department of Medical Technology, Faculty of Associated Medical Sciences, Chiang Mai University, Chiang Mai 50200, Thailand; c Center for Excellence in Pharmaceutical Nanotechnology, Faculty of Pharmacy, Chiang Mai University, Chiang Mai 50200, Thailand; d Department of Cosmetic Sciences, School of Pharmaceutical Sciences, University of Phayao, Phayao 56000, Thailand; e College of Pharmacy, Seoul National University, Seoul 151-741, South Korea

**Keywords:** Curcumin, Epigallocatechin gallate, SIRT1, Anti-aging, HaCaT cells, Nanostructured lipid carriers, Skin

## Abstract

Curcumin (Cur) and epigallocatechin gallate (EGCG), the primary active compounds in turmeric and green tea, respectively, have been investigated for their anti-aging potential. The Cur and EGCG combination was encapsulated in sustained-release nanostructured lipid carriers (NLCs) to enhance their bioactivities and pharmaceutical properties. A significant enhancement in the antioxidant activities of the Cur and EGCG combination was observed at an optimal ratio, as demonstrated by the 2,2-diphenyl-1-picrylhydrazyl radical scavenging assay (118.83 ± 3.78 %), ferric ion reducing antioxidant power assay (217.25 ± 13.45 %), and lipid peroxidation inhibition assay (106.08 ± 12.93 %), compared to Cur alone without compromising the antioxidant activities and total phenolic content of EGCG. This is due to the enhancement of total phenolic content of the combination of 218.83 ± 10.57 %. For anti-aging activities, the combination exhibited stimulation of SIRT1 protein and inhibition of collagenase and elastase of 27.53 ± 0.73 %, 43.70 ± 1.05 % and 51.76 ± 6.52 % compared with that achieved with Cur alone, respectively. The incorporation of the Cur and EGCG combination into NLCs resulted in high entrapment efficiencies of 98.60 ± 0.05 % for Cur and 98.40 ± 0.08 % for EGCG, with corresponding loading capacities of 0.789 ± 0.001 % and 3.935 ± 0.003 %, respectively. When formulated NLCs into an emulgel base, the system demonstrated sustained release profiles over 48 h, with 12.82 ± 0.99 % release of Cur and 63.77 ± 5.76 % release of EGCG. Significant skin retention was also observed after 24 h, with 23.88 ± 1.71 % Cur and 22.79 ± 4.65 % EGCG retained in the skin. Therefore, Cur: EGCG-loaded NLCs in emulgel can deliver the active compounds into the dermis, enhancing skin penetration, sustained delivery, and anti-aging activity superior to each conventional single active compound in topical formulations.

## Highlights

• The optimal Cur: EGCG molar ratio (1:5) demonstrated superior anti-aging efficacy.
• The 1:5 of Cur: EGCG combination enhanced antioxidant activity, upregulated the SIRT1 protein, and reduced cytotoxicity in keratinocytes.
• The NLCs of the combination in emulgel showed sustained release and higher skin accumulation.

## Graphical abstract

**Figure:**
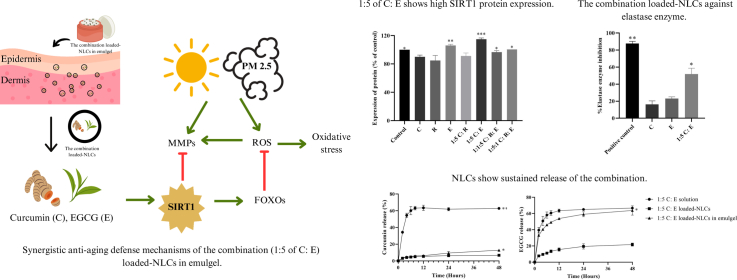

## 1 Introduction

Aging is a process that can happen to all living organisms. Skin deterioration is one of the most observable signs of aging. UV is the most important factor that accelerates skin aging. UV exposure provokes the thickening of the epidermal layer, loss of elasticity, fine wrinkles, and sagging eyelid skin (Sjerobabski-Masnec and Situm, 2010). Sirtuin 1 (SIRT1) is an NAD^+^-dependent deacetylase that regulates physiological processes by modifying protein expression through histone deacetylation. Sirtuins (SIRTs), first discovered as homologs of the silent information regulator 2 (Sir2) in yeast, are extensively studied biomolecules for their potential role in slowing down the aging process (Frye, 1999). SIRT1 protein has been shown to enhance antioxidant and anti-inflammatory properties based on its deacetylase activity, and it regulates gene transcription, chromosome stability, and target protein activity. Overexpression of SIRT1 protein can promote the upregulated activity of antioxidant enzymes, including superoxide dismutase and catalase, mitigating oxidative stress and reducing cell damage (Chung et al., 2015). Recent research has shown that SIRT1 can inhibit some matrix metalloproteinases (MMPs), such as MMP-1 and MMP-3, implicating its influence on intrinsic and extrinsic aging (Lim et al., 2020). In the skin, the MMPs exist in dermal fibroblasts and epidermal keratinocytes. The primary constituents of the extracellular matrix (ECM), hyaluronic acid, elastin, and collagen, are broken down by hyaluronidase, elastase, and collagenase, respectively (Fayad et al., 2018).

Nutritional or pharmacological interventions and lifestyle changes are the key strategies to prolong a healthy lifespan (Liu, 2022). Natural compounds, such as vitamins, polyphenols, hydroxy acids, and polysaccharides, are known to extend longevity and improve quality of life and hence are commonly included in skin care products (Vaiserman et al., 2021; Ainsworth and Gillespie, 2007). For instance, resveratrol, a well-known natural stilbene from grape peels, indicates significant anti-aging potential (Asgary et al., 2018). Furthermore, curcumin and epigallocatechin gallate (EGCG) exhibited strong anti-aging properties in the body and skin by potentiating cellular antioxidant defense (Queen and Tollefsbol, 2010).

Curcumin (Cur), an active compound derived from the turmeric plant (*Curcuma longa* L., Zingiberaceae), has a wide range of pharmacological properties, such as antioxidative, anti-inflammatory, and anticarcinogenic effects. Curcumin demonstrates significant anti-aging effects through various mechanisms, including enhancing of cellular homeostasis, reducing of oxidative stress, and mitigating inflammation. Additionally, its potent antioxidant and anti-inflammatory properties contribute to its role in combating age-related processes, such as apoptosis, autophagy, and cellular senescence (Sharma et al., 2005; Bahrami et al., 2021). However, the disadvantages of turmeric extract, including color staining, poor solubility, low stability, and limited bioavailability, hinder the market availability of Cur as a health product (McClements, 2020). Additionally, allergic reactions and irritation can occur in the skin when directly exposed to Cur (Liu et al., 2022).

Resveratrol (Res) and EGCG are known to inhibit collagenase, which can break down collagen molecules. In addition, both compounds increase cell survival and activate the SIRT1 protein in UV-induced cell death (Masaki, 2010). Either Res or EGCG has been studied for their efficacies when combined with Cur for antioxidant and anticancer activities in cultured prostate cancer cells and a xenograft mouse model (Eom et al., 2015; Narayanan et al., 2009), as well as liver (Du et al., 2013), colon (Patel et al., 2010), and lung cancer cells (Zhou et al., 2013). While most studies on curcumin-antioxidant combinations have focused on cancer preventive or antitumorigenic effects, none addresses skin anti-aging activity. Regarding safety, careful consideration of appropriate doses and ratios of natural product composition is necessary. Res at an excessive concentration (50 μM) was found to induce apoptotic cell death in tumor cells (Shaito et al., 2020). Additionally, EGCG was reported for its instability and irritation at high doses in mice and guinea pigs (Isbrucker et al., 2006). However, appropriate combinations and dosage titration of Cur, Res, and EGCG could minimize toxicity while enhancing anti-aging potential. To overcome the limitations of active compounds derived from plant extracts, such as poor absorption, instability, and potential toxicity, researchers have proposed various strategies. These include utilizing active mixtures and implementing drug delivery systems. In this study, a sustained-release, multi-target formulation combining Cur with EGCG or Res, was developed using a nanostructured lipid carrier (NLCs) delivery system.

NLCs are colloidal drug delivery systems composed of a lipid matrix containing both solid and liquid lipids, designed to encapsulate active compounds—particularly those of natural origin—for sustained release and enhanced skin absorption (Feng et al., 2025). Compared to polymeric or metallic nanoparticles, this lipid-based nanosystem is typically formulated with biocompatible and non-toxic lipids, offering a safe and effective platform for nano-drug delivery (Khan et al., 2023). NLCs can also control the release of active compounds, which can minimize skin irritation (Badalkhani et al., 2023). In pharmaceutical or cosmetic formulations, active-loaded NLCs act as a sustained-release depo for an around-the-clock treatment.

This study investigates the potential application of the combination of Cur with EGCG, or Res as an optimal anti-aging formulation. The anti-aging potential of the combined active compounds was evaluated through their ability to activate SIRT1 protein and inhibit various deleterious processes mediated by reactive oxygen species (ROS) and ECM enzymes. Additionally, the stability, release profiles, and skin retention properties of the extract-loaded NLCs formulation were investigated.

## 2 Material and methods

### 2.1 Plant extract materials

Turmeric extract (95 % purity) was purchased from Welltech Biotechnology, (Bangkok, Thailand). Res (98 % purity) and EGCG (98 % purity) were purchased from MySkinRecipes, (Bangkok, Thailand).

### 2.2 Chemicals

Methanol and ferrous chloride (FeCl_2_) were purchased from RCI Labscan Asia, (Bangkok, Thailand). Acetonitrile was purchased from Macron Fine Chemicals, (VWR International Holdings, Radnor, PA, USA). Folin-Ciocalteu reagent was purchased from Supelco, (Bellefonte, PA, USA). Gallic acid, phosphoric acid, 2,2′-diphenyl-1-picrylhydrazyl (DPPH), Trolox, ferric tripyridyltriazine (TPTZ), linoleic acid, 2,2′-azobis-(2-amidinopropane dihydrochloride (AAPH), hyaluronidase, collagenase, elastase and their substrates and ferrous sulfate were purchased from Sigma-Aldrich, (St. Louis, MO, USA). Dulbecco's Modified Eagle Medium (DMEM), fetal bovine serum (FBS), and penicillin-streptomycin were purchased from Gibco Life Technology, (Thermo Fisher Scientific; Waltham, MA, USA). Glyceryl behenate was purchased from P.C. Intertrade, (Bangkok, Thailand). Cocoa butter was received from MarkRin Chocolate Company, (Chiang Mai, Thailand).

### 2.3 Determination of active compounds in extracts using HPLC analysis

Active compounds in the extracts were analyzed using a modified high-performance liquid chromatography (HPLC) method from Boonrueang et al. (2019). The HPLC system (Agilent Series HP1100) with a UV–visible detector was used with the reversed-phase C-18 column (Purospher® STAR, 5 μm, 150 mm × 4.6 mm), supplied by Merck KGaA (Darmstadt, Germany). A gradient elution was performed using a mobile phase comprising 0.1 % phosphoric acid in water, acetonitrile, and methanol (Table S1). Cur, Res, and EGCG elution was monitored via UV detection at wavelengths of 425, 306, and 278 nm, respectively (Roman, 2013; Singh et al., 2012; Kotra et al., 2019). The flow rate was set at 1.0 mL/min, and the injection volume was 10 μL for all samples and reference standards. The peak areas were calculated to quantify the amounts of active compounds using a calibration curve constructed from 3.125 to 100 μg/mL of standards.

### 2.4 Determination of total phenolic content and antioxidant activity

The total phenolic content and antioxidant activity of individual and combined active compounds derived from plant extracts were evaluated. The molar ratios were calculated based on the active compounds in each extract as determined by HPLC analysis (Table S2). The top four ratios of the combinations that exhibited the highest antioxidant activity and total phenolic content were further investigated for anti-aging activity in cell culture studies.

#### 2.4.1 Determination of total phenolic content

The Folin-Ciocalteu method was used for determining the phenolic content of single and combined active compounds (Ainsworth and Gillespie, 2007). Each sample was prepared at the concentration of 1 mM (25 μL), added to Folin-Ciocalteu reagent (100 μL), and incubated at 25 °C for 4 min. Sodium carbonate solution (75 μL) was added to the mixture. The mixture was incubated in the dark at room temperature for 2 h. The absorbance was measured at 765 nm using a microplate reader (SpectraMax®M3; Sunnyvale, CA, USA). Gallic acid was used as the standard for constructing the calibration curve.

#### 2.4.2 DPPH radical scavenging activity assay

The radical scavenging activity of single and combined active compounds against DPPH radicals was assessed using the method by Brem et al. (2004). The sample at a concentration of 500 μM (20 μL) was added to 180 μL DPPH reagent (167 μM) and incubated in the dark at 25 °C for 30 min. Trolox was used as a positive control. The absorbance was measured at 519 nm and the DPPH radical scavenging activity was calculated by using the following eq. (1).

(1) $$\%\mathrm{inhibition}=\frac{A\;\mathrm{control}-A\;\mathrm{sample}\;}{A\;\mathrm{control}}\times 100$$

Where A_control_ is the absorbance of control, A_sample_ is the absorbance of the active compounds.

#### 2.4.3 FRAP Assay

The FRAP values of single and combined active compounds were determined by the method from Okonogi et al. (2007). FRAP reagent was prepared from TPTZ, ferric chloride, and acetate buffer in a ratio of 10:1:1 (% *v*/v/v). Each sample prepared at the concentration of 1 mM (20 μL) was added to 180 μL FRAP reagent and incubated at room temperature for 5 min. Trolox was used as a positive control. The absorbance was measured at 595 nm using ferrous sulfate as a standard.

#### 2.4.4 Lipid peroxidation inhibition assay

The lipid peroxidation inhibition of single and combined active compounds was determined using the method of Olszewska (2011). Each sample prepared at the concentration of 4 mM (150 μL) was mixed with 350 μL linoleic acid, and 50 μL AAPH in phosphate buffer, pH 7.0, and incubated at 50 °C for 4 h. Then, 5 μL of NH_4_SCN, 5 μL of FeCl_2_, and 185 μL of methanol were added to the mixture (5 μL), followed by incubation at 25 °C for 3 min. Trolox was used as a positive control. The absorbance was measured at 500 nm, and lipid peroxidation inhibition capacity was calculated using the following eq. (1).

### 2.5 Determination of cell viability upon exposure to active compounds

The single compounds and combinations were investigated for the effect on cell viability by the modified MTT assay (Roy et al., 2010). Human keratinocyte HaCaT cells were cultured in DMEM media containing 10 % FBS and 1 % penicillin-streptomycin at 37 °C in 5 % CO_2_ to achieve 80–90 % confluence. Each sample (20 μM) was treated and incubated at 37 °C in 5 % CO_2_ for 48 h. The media were then replaced with MTT solution (15 μL) and further incubated for 4 h. The absorbance was measured at 578 nm with a reference wavelength of 630 nm using the ELISA microplate reader (Metertech, Taiwan, China). The cell viability was calculated from the absorbance values of the test and control using the following eq. (2).

(2) $$\%\mathrm{inhibition}=\left(A_{control}-A_{sample}\right)\times 100$$

Where A_control_ is the absorbance of control, A_sample_ is the absorbance of the active compounds.

### 2.6 Determination of SIRT1 protein expression

The single compounds and combinations were investigated for the effect on SIRT1 protein expression. The HaCaT cells (2.5 × 10^5^ cells/mL) were incubated with the single compounds and combinations at a concentration of 3 μM for 48 h. The cells were harvested and washed before the whole protein extraction process using RIPA buffer. Samples (25 μg protein) were subjected to 7.5 % SDS-PAGE and then transferred to polyvinylidene difluoride (PVDF) membranes. The membranes were blocked for nonspecific binding by 5 % skim milk in phosphate buffer (PBS), pH 7.4, for 2 h at room temperature. Each membrane was incubated with rabbit polyclonal anti-SIRT1 IgG (MilliporeSigma; Missouri, MA, USA) at a dilution of 1:5000 and rabbit polyclonal anti-human GAPDH IgG (MilliporeSigma; Missouri, MA, USA) at a dilution of 1:10,000 for 2 h. Membranes were washed and incubated with HRP-conjugated goat anti-rabbit IgG (Invitrogen™; Carlsbad, CA, USA) at a 1:20,000 dilution for 1 h. Protein bands were detected using Luminata™ Forte Western HRP substrate (Merck; Darmstadt, Germany) and then exposed to an X-ray film (FINE Med; Hebei, China). Band densities were quantitated using the Quantity One 1-D Analysis software (Bio-Rad; Carlsbad, CA, USA).

### 2.7 Determination of SIRT1 protein deacetylase activity

SIRT1 deacetylase activity was measured using a commercial kit (Sirt1 Assay Kit CS1040, Sigma-Aldrich; St. Louis, MO, USA) following the manufacturer's protocol. The fluorescence signal was measured at excitation/emission wavelengths of 355/460 nm using a microplate reader (Lamichane et al., 2019). Finally, the SIRT1 protein deacetylase activity was calculated by comparing it with a standard curve.

### 2.8 Development of NLCs

The selection of solid and liquid lipids was screened preliminarily using the compatibility test between active compounds and the lipid phase. The systems that provided optimal compatibility were further optimized using the compositions illustrated in Table S3. The combination of active extracts entrapped in the NLCs was selected based on bioactivity tests, which included strong antioxidant activity and SIRT1 protein expression.

#### 2.8.1 Preparation of unloaded NLCs and combination-loaded NLCs

The NLCs were developed using high-shear homogenization, which was modified by the method of Rahman et al. (2013). The formulation of NLCs was optimized by adjusting the proportions of solid lipids (glyceryl behenate, cetearyl alcohol, or cocoa butter) and liquid lipids (krill oil, meadowfoam seed oil, or sweet almond oil) to achieve enhanced skin penetration properties. The optimized NLCs should exhibit a particle size range of 50–300 nm, a narrow polydispersity index (PDI < 0.3), and a stable zeta potential (±30 mV) (Ghasemiyeh and Mohammadi-Samani, 2018; Tamjidi et al., 2013; Loo et al., 2013). A liquid lipid was selected from the antioxidant activity using the DPPH assay. The optimal particle size, PDI, and stability of NLC formulations for dermal application were determined. The ingredients from NLCs are shown in Table S3. The combination (0.12 %, *w*/w) was dissolved in a melted lipid mixture and Span® 80 under controlled stirring at 75 °C. An aqueous phase was prepared by mixing distilled water with Tween® 80 and heated to 80 °C. The water phase was added to the lipid phase and mixed using a high-shear homogenizer (T 25 digital ULTRA-TURRAX®; Staufen, Germany) at 15,000 rpm for 10 min. The combination-loaded NLCs were cooled to room temperature. The characterization of NLCs, including particle size, PDI, and zeta potential, was investigated via a Zetasizer ZS (Malvern Instruments; Worcestershire, UK) following the method of Anantaworasakul et al. (2020).

#### 2.8.2 Stability of NLCs

The unloaded NLCs were formulated as described in Table S3. The nanoparticles’ suitability for transdermal delivery and stability was characterized. The unloaded NLCs were stored in well-closed glass bottles and kept at 30 °C for 7 days. The samples were centrifuged at 3000 ×*g* for 20 min. The particle size and PDI at Day 0, Day 7, and after centrifugation were monitored using the Zetasizer. The formulations that possessed desirable properties were selected for further combination entrapment.

The storage stability of the developed combination-loaded NLCs was assessed by monitoring changes in particle characteristics under various conditions. The samples were stored in amber glass bottles at 4 °C, 30 °C (light and dark), 45 °C for 28 days, and 6 heating-cooling cycle conditions (heating-cooling 1 cycle: at 4 °C for 48 h was followed by 45 °C for 48 h.). Additionally, the stability of the combination-loaded NLCs in the emulgel was assessed under various conditions, as previously described. After storage, the pH value and antioxidant activity of the combination-loaded NLCs in the emulgel were measured and compared with their initial values.

#### 2.8.3 Determination of entrapment efficiency (EE) and loading capacity (LC)

The combination-loaded NLCs suspension was pipetted into the upper chamber of Amicon® ultra 0.5 mL centrifugal filters (Amicon®-50 k, Merck; Darmstadt, Germany) and centrifuged at 15,652 ×*g* for 10 min. The solution in the lower chamber was diluted at a ratio of 1:5 of solution and methanol. The dilutions were filtrated and analyzed using HPLC. The percentages of EE and LC were calculated using eqs. (3), (4), respectively.

(3) $$\%\mathrm{EE}=\frac{\text{Initial concentration of active compound}–\text{active compound concentration in filtrate}}{\text{Initial concentration of active compound}}\times 100$$

(4) $$\%\mathrm{LC}=\frac{\text{weight of active compound in lipid nanoparticles}\;}{\text{weight of lipid nanoparticles}}\times 100$$

### 2.9 Physicochemical and Bioactivity Properties of Combination-loaded NLCs

#### 2.9.1 Determination of Morphology using Transmission Electron Microscopy (TEM)

The morphology of the combination-loaded NLCs was determined using transmission electron microscopy (TEM, JEM-2011; JEOL, Tokyo, Japan). NLCs were diluted with deionized water at a ratio of 1:100. A drop of the diluted sample was placed onto a copper grid, dried, and stained with 2 % phosphotungstic acid. The grid was then allowed to dry at room temperature. TEM analysis was performed at 100 kV.

#### 2.9.2 Determination of anti-aging activity

##### 2.9.2.1 Hyaluronidase inhibitory activity assay

The anti-hyaluronidase activity of individual compounds and their combinations was evaluated using a modified spectrophotometric assay. The samples at a concentration of 0.84 mg/mL (50 μL) were mixed with 100 μL hyaluronidase (2.5 mg/mL) and incubated at 37 °C for 10 min. The mixture was added to 100 μL hyaluronic acid (0.003 % *w*/*v*) and incubated at 37 °C for 45 min. Acetic albumin dilution (1000 μL) was added to the mixture and incubated at 25 °C for 10 min to stop the reaction. The absorbance was measured at 600 nm using a microplate reader. Hyaluronidase inhibitory activity was calculated using the eq. (2). Tannic acid was used as a positive control (Piwowarski et al., 2011).

##### 2.9.2.2 Collagenase inhibitory activity assay

The anti-collagenase activity of single compounds and combinations was investigated by spectrophotometric assay with some modification. The collagenase solution and the N-{3-(2-Furyl)acryloyl}-L-leucine-glycyl-L-propyl-L-alanine (FALGPA) were prepared and dissolved in a 50 mM Tricine buffer (pH 7.5). The samples at a concentration of 0.84 mg/mL (10 μL) and 40 μL of collagenase solution (2 mg/mL) were added to 96 well plates. Then, the mixtures were incubated at 25 °C for 15 min, followed by the addition of 50 μL FALGPA solution (0.5 mg/mL). The absorbance was measured at 340 nm using a microplate reader. Collagenase inhibitory activity was calculated using the eq. (1). Standard EGCG was used as a positive control (Thring et al., 2009).

##### 2.9.2.3 Elastase inhibitory activity assay

The anti-elastase activity of single compounds and combinations was investigated by spectrophotometric assay with some modification. The elastase solution and 4.4 mM N-Succinyl-L-alanyl-L-alanyl-L-alanine 4-nitroanilide (AAAPVN) were prepared and dissolved in a 100 mM Tris-HCl buffer (pH 8.0). The samples at concentration of 0.84 mg/mL (50 μL), and 25 μL elastase solution (8 μL/mL) were added to 96 well plates and incubated at 25 °C for 20 min. AAAPVN (25 μL) was added to the mixture. The absorbance was measured at 410 nm using a microplate reader. Elastase inhibitory activity was calculated using the eq. (1). Standard EGCG was used as a positive control (Thring et al., 2009).

### 2.10 Development of Emulgel base from combination-loaded NLCs

#### 2.10.1 Preparation of emulgel

Emulgel was prepared using a beaker method. Avocado oil, rice bran oil, jojoba oil, and tocopherol acetate (2 % each), together with a sorbitan ester–polysorbate emulsifier system (10 %), were used in the oil phase of the emulsion system. The aqueous phase, along with 5 % glycerin and 1 % phenoxyethanol, was heated to the same temperature as the oil phase (70–75 °C). A 1.5 % gelling agent (Carbopol 940®) was dispersed in water and neutralized to form a gel base, into which the emulsion was gradually incorporated and stirred to form a homogeneous emulgel. The emulgel and combination-loaded NLCs were homogeneously mixed at a ratio of 1:1. The final formulation was evaluated for homogeneity, viscosity, and stability under various conditions before storage.

#### 2.10.2 In vitro release of actives from the formulations

In vitro release ability of actives was evaluated using a dialysis method. The release of actives from the combination solutions, the combination-loaded NLCs, and the combination-loaded NLCs in emulgel (5 g) was monitored by using a cellulose dialysis membrane tubing (Cellu•Sep® T4, Membrane filtration Product; Seguin, TX, USA) with 12,000–14,000 kDa molecular weight cut off. The membrane was previously hydrated for 24 h., filled with the samples, and immersed in the medium consisted of 2:8 in methanol: 100 mM PBS buffer, pH 5.5 (60 mL) at 32 °C with continuous stirring. Samples (1 mL) were collected and replenished at 0, 2, 4, 6, 8, 12, 24, and 48 h, filtered, and measured for the active compounds using a UV–vis spectrophotometer at 278 and 425 nm for EGCG and Cur, respectively. Moreover, samples were studied for antioxidant activity using the DPPH method.

#### 2.10.3 Determination of skin penetration

Skin penetration of the combination loaded in the NLCs and the NLCs in emulgel was performed in comparison with the solution and the untrapped counterparts using the Franz diffusion method (Chen et al., 2012). Strat-M® membrane (25 mm, Merck Millipore Ltd.; County Cork, Ireland) was used as a synthetic skin membrane for transdermal prediction. Each sample (1 g) was applied to the membrane surface. The receptor chamber (12 mL) was filled with medium consisting of 2:8 methanol: PBS (pH 5.5) with the temperature control of 37 °C. Aliquot of penetrated solution (1 mL) was collected at 1, 2, 4, 6, 8, 12, and 24 h from the receptor compartment with the fresh medium replacement. After 24 h, samples in the Strat-M® membrane were extracted from the membranes using 2 mL of methanol. All aliquots were analyzed by HPLC as previously mentioned.

### 2.11 Statistical analysis

Data were reported as the mean ± standard deviations. Statistical significance was determined by one-way ANOVA using the SPSS program, version 17.0 (IBM SPSS, Armonk, NY, USA), and *p*-values <0.05 were considered statistically significant.

## 3 Results and discussion

### 3.1 Active compounds in plant extracts

**Fig. 1:**
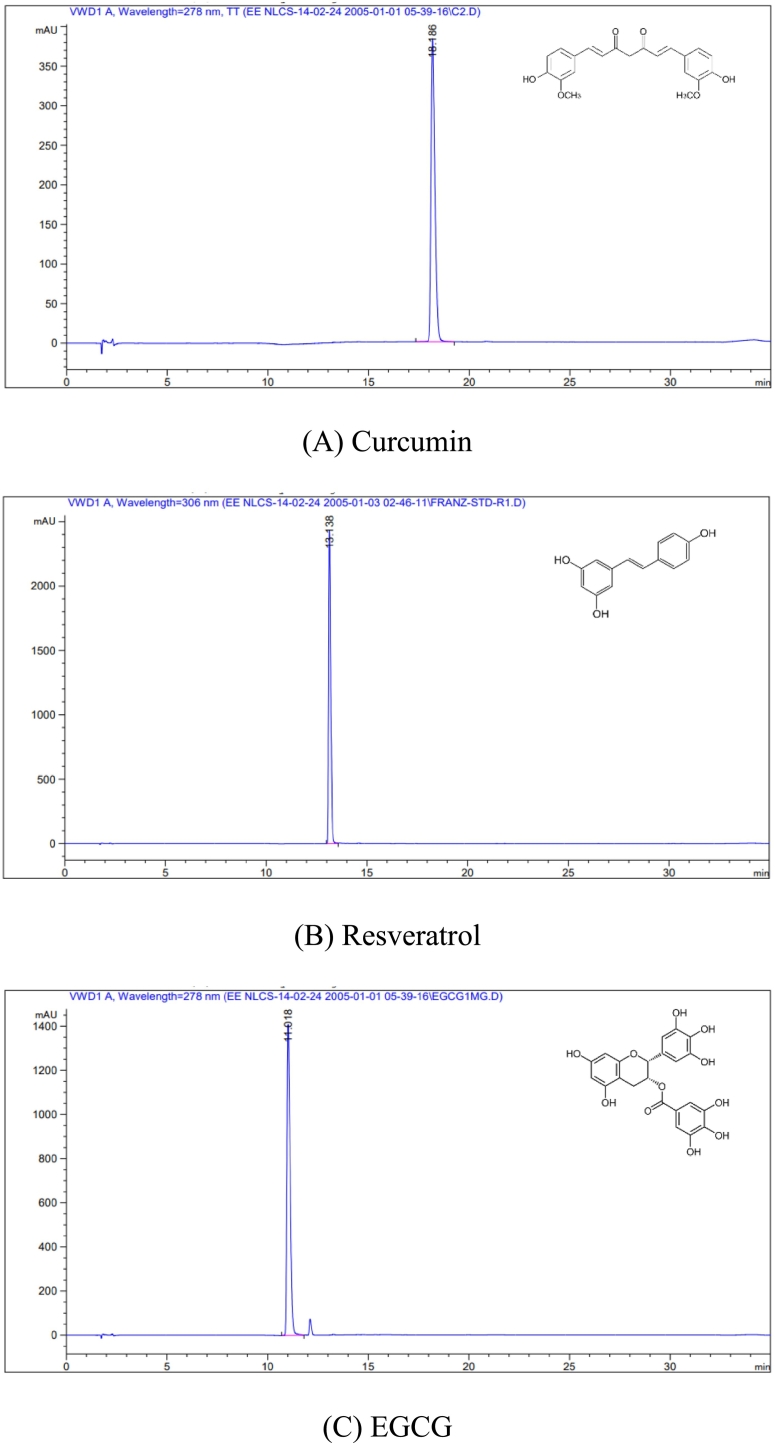

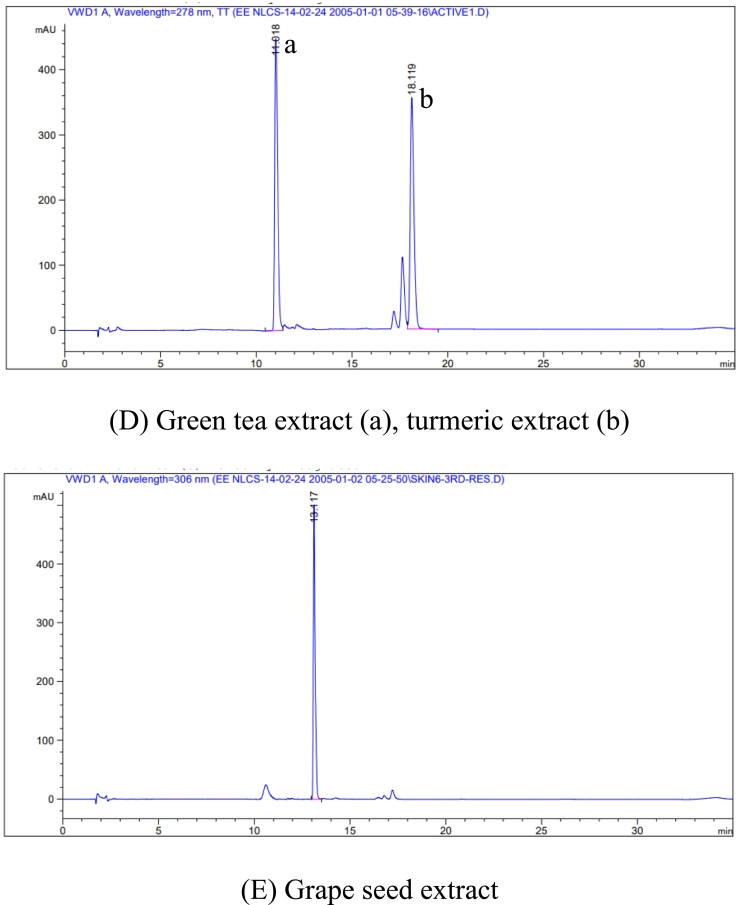
HPLC chromatograms of **(**A) Cur detected at 425 nm, (B) Res detected at 306 nm, (C) EGCG detected at 278 nm, (D) green tea extract and turmeric extract detected at 278 and 425 nm, respectively, and (E) grape seed extract detected at 306 nm. (For interpretation of the references to color in this figure legend, the reader is referred to the web version of this article.)

The active compound content in the extracts is a major parameter for the determination of the molar ratios of the mixtures. The HPLC chromatograms revealed that the turmeric, grape seed, and green tea extracts contained Cur, Res, and EGCG as the major compounds (Fig. 1A-E). Turmeric extract (Fig. 1D(b)) contained Cur at 66.45 ± 2.88 % (retention time: 18.1–18.2 min), representing the major active compound. Fig. 1B and E display the chromatograms of Res standard and grape seed extract, which contained Res as the only main active compound with a percentage of 83.95 ± 7.46 at a retention time of 13.1 min (Fig. 1E). Finally, Fig. 1C and D(a) show the EGCG peak of the EGCG standard and the green tea extract at a retention time of 11.0 min, which is the main active compound with a percentage of 83.69 ± 2.10. The analytical method using the modified HPLC protocol allowed the analysis of all active compounds, and no interference was observed in the study. In addition, these extracts were proven to contain the highest commercially available active compounds, which were subsequently combined with various molar ratios for bioactivity evaluation (Table S2).

### 3.2 Total phenolic content and antioxidation activity

The total phenolic content and antioxidation activity of the 10 active compound combinations are shown in Table 1. These combinations were combined at various ratios with the total concentration was equivalent. Total phenolic content of the combinations, as shown in Table 1 containing EGCG as a combination at the ratios of 1:5 Cur: EGCG, 1:10 Cur: EGCG, 1:1:5 Cur: Res: EGCG, and 1:5:5 Cur: Res: EGCG had significantly higher total phenolic content when compared with other samples at the same concentration. According to data from the DPPH scavenging activity assay, all combinations containing EGCG except the ratio of 1:5:1 Cur: Res: EGCG exhibited the percentages of inhibition significantly higher (approximately 89 %) than that of Trolox (72.72 ± 4.97 %), positive control at the same concentration as shown in Table 1 (*p* < 0.001). The results from the FRAP assay are shown in Table 1. All combinations between Cur and EGCG except the ratio of 1:1 Cur: EGCG exhibited FRAP values significantly higher than other samples, approximately 2500 M FeSO_4_/ mol total active compounds (*p* < 0.0001). This value is also significantly higher than the value from Trolox, which yielded the FRAP value of 1437.93 ± 69.84 M FeSO_4_/ mol total compound at the same concentration. The results of the lipid peroxidation inhibition study are presented in Table 1. All combinations of Cur and EGCG together with Cur: Res: EGCG (1:1:1) showed the percentages of inhibition significantly higher, approximately 60 % (*p* < 0.01) compared to other combinations at the same concentration. The results relate to the values from the previous study in which green tea, combined with grape seed, gooseberry, pomegranate, cinnamon, and ginkgo extract, showed synergistic antioxidant activity compared to green tea alone (Jain et al., 2011). In a total equimolar comparison, among the three active compounds, EGCG from green tea exhibited the strongest antioxidant, followed by Cur and Res in all antioxidant assays. All tested active compound mixing ratios containing EGCG could enhance the antioxidant activity of Cur. Cur combined with EGCG increased the antioxidant activity of glutathione (GSH) and superoxide dismutase (SOD), diminishing oxidative stress more effectively compared to single active compounds (Rahman et al., 2023). The antioxidant activity of the combinations in all three different mechanisms was related to the total phenolic content in the active compounds. The higher total phenolic content affected the antioxidation effect (Dobrinas et al., 2021; Velioglu et al., 1998). The phenolic hydroxyl groups in the structures play a key role in the capacity to scavenge free radicals, reducing the oxidized moieties and inhibiting lipid peroxidation. The number and position of hydroxyl groups and other groups influence antioxidant activity (Bors et al., 2001). Cur, Res, and EGCG have 2, 3, and 8 hydroxy groups, respectively. In this experiment, EGCG showed the strongest antioxidant activity than Cur and Res which is comparable to the activity obtained from the Cur: EGCG combination. Therefore, the 1:5 of Cur: EGCG and 1:1:5 of Cur: Res: EGCG were selected to evaluate anti-aging activity, in comparison with 1:5 of Cur: Res and 1:5:1 of Cur: Res: EGCG because Res is a key compound known for its anti-aging efficacy (Leis et al., 2022). According to the free radical theory of aging, excessive reactive oxygen species (ROS) contribute to age-related deterioration by inducing oxidative stress, inflammation, and cellular damage. ROS are continuously generated as metabolic by-products, particularly from mitochondrial respiration, and their levels are further exacerbated by environmental factors such as UV radiation and pollution. Antioxidants scavenge ROS by donating electrons, thereby protecting cellular components from intracellular damage. Potent plant-derived antioxidants, including ascorbic acid, resveratrol, epigallocatechin gallate (EGCG), and curcumin, have demonstrated efficacy in mitigating oxidative damage (Petruk et al., 2018; Pandel et al., 2013). Excessive ROS generation is associated with various cellular processes, including the release of pro-inflammatory cytokines, upregulation of collagenase, direct oxidation of DNA, and indirect modifications of DNA, proteins, and lipids. Sirtuin 1 (SIRT1), a protein involved in skin aging, plays a crucial role in regulating these processes, and its activation can counteract ROS-induced damage (Bosch et al., 2015). Thus, antioxidants not only protect against oxidative stress but also modulate key longevity pathways, including SIRT1 activation, ultimately supporting skin health and overall longevity. Both dietary and topical antioxidant supplementation may serve as effective strategies for preserving youthful cellular function (Sadowska-Bartosz and Bartosz, 2014).

**Table 1:** Total phenolic content and antioxidant activity of samples and standards from three different methods: DPPH assay, FRAP assay, and lipid peroxidation inhibition assay.

Sample	Methods			
	TPC (mM GA/ mol compound)	DPPH activity (%inhibition)	Reducing capacity (FRAP assay, M FeSO_4_/ mol total compound)	Lipid peroxidation inhibition activity (%inhibition)
Trolox (positive control)	–	72.72 ± 4.97**	1437.93 ± 69.84*	66.80 ± 4.27**
Cur	0.72 ± 0.03	76.13 ± 2.29**	903.77 ± 58.17	54.06 ± 1.56
Res	0.81 ± 0.03	38.31 ± 2.56	836.92 ± 83.10	31.30 ± 5.71
EGCG	1.57 ± 0.03**	89.56 ± 0.64***	2741.45 ± 154.54****	69.46 ± 3.95**
1:1 Cur: Res	0.78 ± 0.02	63.79 ± 3.41*	903.13 ± 50.13	61.92 ± 3.38**
1:5 Cur: Res	0.85 ± 0.03	43.75 ± 0.84	943.36 ± 60.60	54.15 ± 5.32
1:10 Cur: Res	0.92 ± 0.03	41.45 ± 1.76	894.71 ± 56.09	51.28 ± 2.97
1:1 Cur: EGCG	1.36 ± 0.05*	89.47 ± 0.19***	1864.15 ± 157.18**	62.09 ± 5.36**
1:5 Cur: EGCG	1.57 ± 0.02**	89.84 ± 0.48***	2522.08 ± 9.11****	60.80 ± 3.72**
1:10 Cur: EGCG	1.59 ± 0.05**	89.56 ± 0.16***	2614.28 ± 111.47****	62.98 ± 9.17**
1:1:1 Cur: Res: EGCG	1.34 ± 0.06*	89.01 ± 0.11***	1447.95 ± 59.92*	63.39 ± 5.66**
1:5:1 Cur: Res: EGCG	1.13 ± 0.01	71.98 ± 4.25**	1324.07 ± 90.24*	56.27 ± 5.48
1:1:5 Cur: Res: EGCG	1.75 ± 0.05***	89.42 ± 0.05***	2267.58 ± 159.93***	59.64 ± 4.34*
1:5:5 Cur: Res: EGCG	1.49 ± 0.07**	89.21 ± 0.13***	1793.59 ± 90.25**	60.02 ± 5.48**

Results are shown as mean ± S.D. (*n* = 3). * p < 0.05, ** p < 0.01, *** p < 0.001, **** p < 0.0001 denoted significant difference samples.

### 3.3 Effect of the combination on SIRT1 protein

#### 3.3.1 Effect of active compound combinations on HaCaT cell viability

**Fig. 2:**
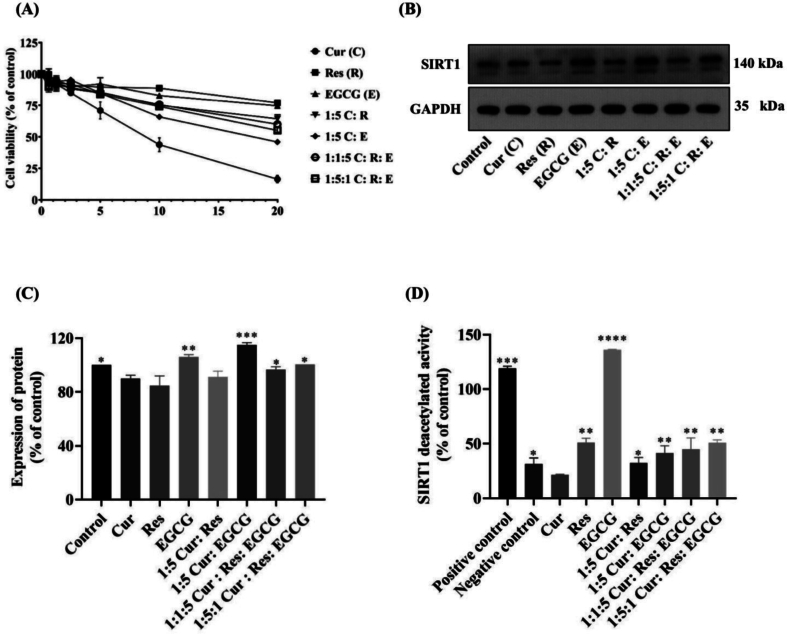
Effect of Cur, Res, EGCG, and combinations on cytotoxicity and SIRT1 protein expression in HaCaT cell line. (A) Cell viability of HaCaT keratinocyte cell line after treatment with Cur, Res, EGCG, and the combinations (1:5 Cur: Res, 1:5 Cur: EGCG, 1:1:5 Cur: Res: EGCG, and 1:5:1 Cur: Res: EGCG) for 48 h. (B) Protein expression of SIRT1 from HaCaT cells after the treatments with 3 μM single and combinations of active compounds using Western blot analysis. (C) The normalized SIRT1 protein expression is expressed as percentages of the non-treated control cells using the Quantity One Software (Bio-Rad). (D) SIRT1 deacetylase activity was performed using a commercially available kit. Measurement of SIRT1 deacetylase activity along with the positive control (Res) are expressed as percentages of the vehicle control. Results are shown as mean ± S.D. (*n* = 3). Significant differences compared to data obtained with the treatment of all samples are identified with * p < 0.05, ** p < 0.01, *** p < 0.001, **** p < 0.0001*,* respectively.

HaCaT cell line, an immortalized human keratinocyte cell line, is commonly used to predict skin irritation from the cell line (Deyrieux and Wilson, 2007). The HaCaT cell viability after exposure to combinations of active compounds is shown in Fig. 2A. Although Cur was widely accepted for its effective anti-inflammatory and antioxidant activities (Liu et al., 2022). It was toxic to the HaCaT cells with an IC_50_ value of 9.05 ± 0.65 μM. The combination of a ratio of 1:5 Cur and EGCG (IC_50_ value of 17.97 ± 0.72 μM) had significantly reduced cytotoxicity (*p* < 0.05). Other combinations (such as 1:5 Cur: Res, 1:1:5 Cur: Res: EGCG, and 1:5:1 Cur: Res: EGCG) were not toxic at a concentration of 20 μM. Cur was combined with Res or EGCG. Res and EGCG can reduce the cytotoxicity of Cur. Cur has high toxicity to cell lines, with an IC_50_ value of approximately 10 μM (Zhou et al., 2013; Zhou et al., 2021).

#### 3.3.2 Activation of SIRT1 protein in HaCaT cells by the selected combinations

Nontoxic concentrations (< IC_20_ value) from the cell viability study were employed for the SIRT1 protein modulation investigation after treatment of HaCaT cells for 48 h, as shown in Fig. 2B and C. The ratio 1:5 of Cur: EGCG (114.80 ± 0.63 %) showed a significant increase of SIRT1 protein expression when compared to other treatments (*p* < 0.001*)*. Res as a positive control showed 84.92 ± 2.38 % of SIRT1 protein expression. The SIRT1 protein expression of 1:5 of Cur: Res was 91.29 ± 1.43 %. The combination of Cur and EGCG at a ratio of 1:5 showed a higher percentage of SIRT1 expression than Res and 1:5 of Cur: Res. The combination of Cur, Res, and EGCG at ratios 1:1:5 and 1:5:1 showed SIRT1 protein expression of 96.77 ± 0.70 % and 100.40 ± 0.01 %, respectively. The result from the previous study demonstrated that EGCG at a concentration of 10 μM could induce SIRT1 expression up to 140 % compared with control (100 %) in SH-SY5Y cells (Lee et al., 2015).

Overexpression of the SIRT1 protein could enhance oxidative stress resistance via FOXO 3α promoted upregulation of the superoxide dismutase 2 (SOD2) and catalase, enzymes involved in antioxidant activity (Chung et al., 2015). SIRT1 protein activators were able to reduce apoptosis and promote cell survival in tumor cell lines (Lim et al., 2020). Reduction of antioxidant activity and apoptosis from the overexpression of SIRT1 protein was the result of prolonged cellular senescence or anti-aging. A combination of Cur and EGCG at the ratio of 1:5 could enhance SIRT1 protein expression. This combination can be used as anti-aging skin products or supplements.

#### 3.3.3 Effect of the combination on SIRT1 protein deacetylase activity

The activity of the combination on the SIRT1 deacetylation is shown in Fig. 2D. EGCG alone (226.50 ± 2.81 % of control) exhibited the significantly highest activity (*p* < 0.0001), followed by Res (145.00 ± 2.03 % of control, *p* < 0.001), a positive control compared to the other samples. All combinations showed significantly higher SIRT1 activity (*p* < 0.001), except 1:5 of Cur: Res showing SIRT1 activity in approximately 140 % of control. Res has been reported to increase SIRT1 activity using SIRT1 assay kit in a rat model (Jian et al., 2014). SIRT1 is a nicotinamide adenine dinucleotide (NAD^+^)-dependent deacetylase enzyme that regulates several crucial proteins, including p53, FOXO, PGC-1α, and NF-κB, which are integral to regulating apoptosis, metabolism, and inflammation (Kalous et al., 2016). The deacetylation activity of SIRT1 can be enhanced by Res, a well-established natural SIRT1 activator. Activators enhance the interaction between SIRT1 and its substrates by stabilizing the enzyme or facilitating substrate binding (Jian et al., 2014).

### 3.4 Pre-formulation of NLCs

**Fig. 3:**
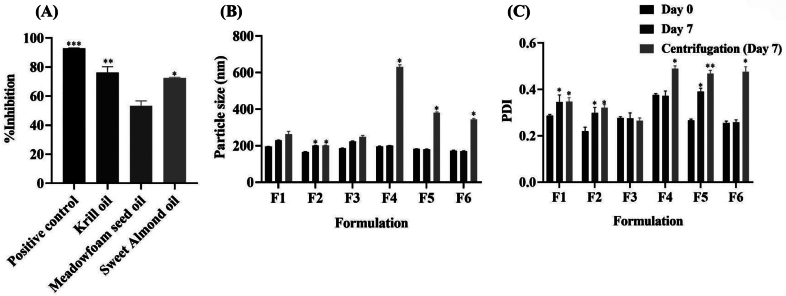
Pre-formulation of NLCs*,* (A) the antioxidant activity of liquid lipids evaluated by DPPH assay. Trolox was used as a positive control. * p < 0.05, ** p < 0.01 and *** p < 0.001 indicate significance level compared with all samples. (B) Particle size and (C) PDI of the unloaded NLCs before and after the stability test. * *p <* 0.05, ** p < 0.01 compared between before and after stability test (*n* = 3).

Before preparing the NLCs, the antioxidation effect of the liquid lipid was tested using the DPPH method, as shown in Fig. 3A. Krill oil (76.27 ± 2.34 %) and sweet almond oil (72.57 ± 0.25 %) showed significantly higher DPPH inhibition than that from the meadowfoam seed oil (53.29 ± 1.95 %) at *p* < 0.05. Krill oil, rich in omega-3 fatty acids, is known for its potent antioxidant effects. The omega-3 polyunsaturated fatty acids in krill oil contribute to anti-obesity, anti-diabetic, anti-cancer, anti-inflammatory, and antioxidant activities (Xie et al., 2019; Colletti et al., 2021). Furthermore, sweet almond oil is a rich source of vitamin E and has high antioxidation properties. It was used as a moisturizer and lubricant in cosmeceutical products, preventing drying and allergies to the skin (Kamkaen et al., 2017). The sweet almond oil was found to possess high stability and antioxidant activity during a 12-months storage period (Bernoussi et al., 2024). Consequently, this plant-origin oil was selected for further NLCs development. During the preformulation stage, several considerations were addressed. Cur and EGCG have low molecular weights of 368.38 g/mol and 458.372 g/mol, respectively, with log *P* values of 2.3–3.2 and 1.0 (Heger et al., 2013; Rosita et al., 2019). Compounds capable of penetrating the skin generally exhibit molecular weights of 500 Da or less (Bos and Meinardi, 2000) and optimal partition coefficients (log P) between 1 and 3. Despite meeting these criteria, limited skin penetration of Cur and EGCG was observed in this study. Enhancing skin penetration using nanostructured lipid carriers (NLCs) has gained significant attention. Optimal blending of liquid and solid lipids in NLC formulations can achieve high entrapment efficiency of active compounds and effective interaction with the skin (Wani et al., 2020). The solubility of Cur and EGCG in various oils was evaluated to identify the oil providing the highest solubilization capacity (Table S4). Sweet almond oil was selected for the final NLC fabrication. Incorporating optimal oils into NLCs promotes desirable nanoparticle morphology and enhances skin penetration. This is achieved through an occlusive effect, which reduces trans-epidermal water loss and forms a film on the stratum corneum surface, thereby improving nanoparticle adherence and increasing the quantity and depth of skin penetration. Additionally, the ability of oils to interact with the lipid bilayer and induce lipid rearrangement may further facilitate the penetration of encapsulated molecules (Varrica et al., 2021).

The unloaded NLC formulations prepared by high-shear homogenization were optimized and characterized, as shown in Table 2. Uniform nanoparticles, typically ranging in size from 50 to 300 nm with a narrow PDI (less than 0.30), were reported to enhance skin penetration (Ghasemiyeh and Mohammadi-Samani, 2018; Tamjidi et al., 2013). The F1-F3 formulations exhibited sizes ranging from 160 to 200 nm and were compared for stability over 7 days. Fig. 3B and C show the stability of the unloaded F1-F6 NLCs. The F3 NLCs exhibited the greatest stability in terms of particle size (140–160 nm) and PDI (less than 0.3) consistency upon storage for 7 days and after centrifugation. While the other formulations exhibited increased particle sizes (Fig. 3B) and PDI (Fig. 3C) (*p* < 0.05) after the stability tests. Therefore, F3 was selected for further study.

**Table 2:** Particle size and PDI of different unloaded NLC formulations. Results are shown as the means ± S.D. (n = 3).

Unloaded NLCs	Particles size (nm)	PDI
F1	194.17 ± 1.88	0.28 ± 0.00
F2	165.97 ± 1.58	0.22 ± 0.02
F3	184.90 ± 2.25	0.26 ± 0.01
F4	360.13 ± 11.05	0.78 ± 0.04
F5	244.00 ± 4.69	0.46 ± 0.01
F6	268.70 ± 12.52	0.46 ± 0.02

### 3.5 Characterization, morphology, and stability of the combination-loaded NLCs

The 1:5 of Cur: EGCG combination was incorporated at 0.12 % *w*/w into the F3 formulation. The optimized combination-loaded NLCs had satisfactory physicochemical characteristics, namely particle size of 141.20 ± 3.70 nm, a uniform distribution of particle sizes (PDI of 0.21 ± 0.02), which fall in the range of effective dermal nano-delivery carriers (Ghasemiyeh and Mohammadi-Samani, 2018; Tamjidi et al., 2013). The zeta potential is −31.30 ± 0.23 mV, which aligns to greater or lower than ±30 mV, indicating a range conducive to stable NLCs (Loo et al., 2013).

The entrapment efficiencies (EE) of Cur and EGCG in 1:5 of Cur: EGCG-loaded NLCs were 98.60 ± 0.05 and 98.40 ± 0.08 %, respectively. Loading capacities (LC) of Cur and EGCG were 0.789 ± 0.001 % and 3.935 ± 0.003 %, respectively. NLCs are colloidal drug delivery devices at the nanosystem cored by a lipid combination of liquid and solid lipids. Additional space is available for the lipophilic compounds to be incorporated into these nanocarriers because the lipophilic molecules, such as the curcuminoids group (log *P* = 2.3–3.2, Heger et al., 2013), dissolve more readily in liquid lipids and the imperfection structure of the lipid core (Borges et al., 2020). Unlike Cur, EGCG has a more hydrophilic structure (log *P* = 1.0, Rosita et al., 2019) and less compatibility with the lipid core (Zhong et al., 2012). Nevertheless, the LC of EGCG was more than that of Cur because this compound could be trapped in the larger interfacial layer of the oil-in-water emulsion (Aditya et al., 2015), and the amount of EGCG loaded in NLCs was greater than that of Cur. For the entrapment of more hydrophilic moieties, high encapsulation efficiency can be also attributed to interactions between EGCG and solid/liquid lipid molecules within the lipid matrix, facilitated by emulsifiers. Similar findings have been reported for the entrapment of hydrophilic compounds, such as isoniazid (log *P* = −0.402), which demonstrated high entrapment efficiency (84.0 %) in solid lipid nanoparticles (SLNs) composed of Compritol 888 ATO® and stearic acid. **Fourier transform infrared spectroscopy** (FTIR) and differential scanning calorimetry (DSC) studies confirmed favorable interactions between isoniazid and lipid molecules. The interaction between hydrophilic drugs and lipid components is essential, as these compounds may form hydrogen bonds or other molecular interactions that influence their localization and release profile within the lipid-based nanocarrier system (Bhandari and Kaur, 2013). The optimized composition of the solid and liquid lipids, as well as the emulsifier, ensures that the structure of NLCs has enough space to entrap active compounds both lipophilic and hydrophilic characters (Souto et al., 2004). Collectively, the NLCs formulation from this research has the potential for further pharmaceutical and cosmetic applications.

**Fig. 4:**
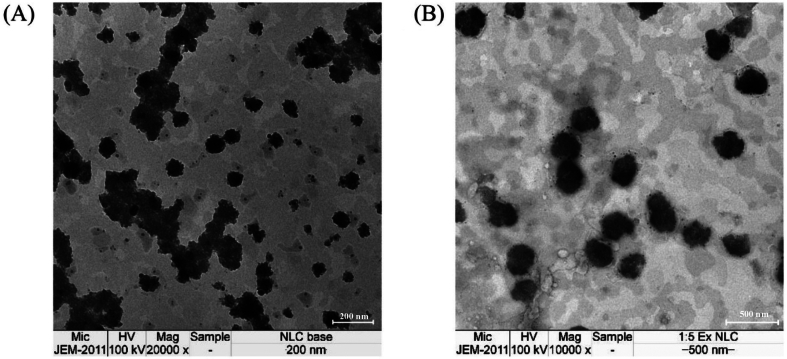
Transmission electron microphotographic images of (A) Blank NLCs and (B) 1:5 of Cur: EGCG-loaded NLCs.

The morphology of the blank NLCs and 1:5 of Cur: EGCG-loaded NLCs were spherical and homogeneously yellowish-colored, with the particle size distributed approximately 200 nm, as shown in Fig. 4. The morphology of the 1:5 of Cur: EGCG-loaded NLCs as shown in Fig. 4A was similar to the blank NLCs as shown in Fig. 4B.

**Fig. 5:**
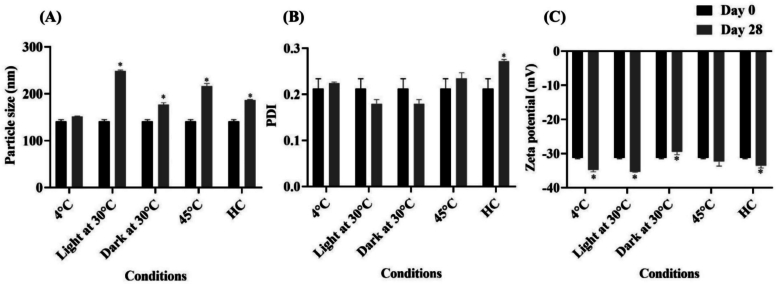
Stability study of combination active compounds loaded NLCs (A) Particle size, (B) PDI, and (C) zeta potential before and after stability test (*n* = 3). * *p* < 0.05 compared between before and after storage stability test. The abbreviation HC stands for heating-cooling condition.

For the stability study, the 1:5 of Cur: EGCG-loaded NLCs were kept at various conditions, including heating-cooling for 6 cycles, 30 °C under light and dark conditions, 4 °C, and 45 °C for 28 days. Physical appearance, particle size, PDI, and zeta potential were analyzed before and after the stability test. After 30 °C and 45 °C storage for 28 days, the color of 1:5 of Cur: EGCG-loaded NLCs became darker than the initial color. In addition, the particle size and zeta potential significantly increased (*p* < 0.05) under HC, 30 °C, and 45 °C conditions. The stability of the NLCs was protected when the samples were stored in 4 °C, as shown in Fig. 5A and C. Interestingly, PDI did not change after storage at 4 °C, 30 °C, and 45 °C, as shown in Fig. 5B. Zeta potential significantly increased (*p* < 0.05) after exposure to 4 °C, light at 30 °C, and HC. High temperatures affected the stability of 1:5 of Cur: EGCG. NLCs due to the core lipid's tendency to melt and transition into a crystalline structure, lead to changes in particle size and PDI (Hu et al., 2006a, Hu et al., 2006b). Cur and EGCG are known for their sensitivity to light (Mondal et al., 2016; Huang et al., 2019). Accordingly, the combination-loaded NLCs should be processed into finished products and stored away from light and high temperatures.

### 3.6 Anti-aging activity of the combination-loaded NLCs

**Fig. 6:**
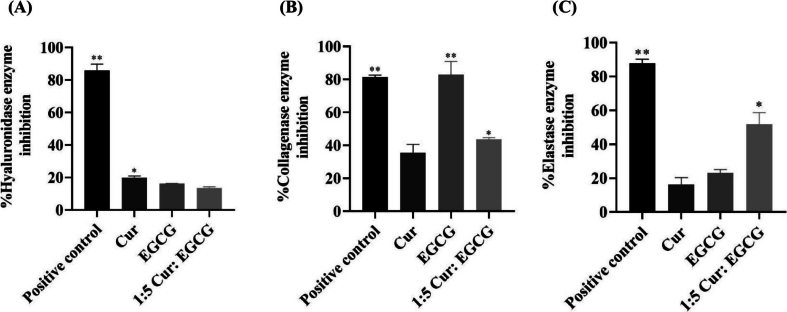
Potential of active compounds and the combination against hyaluronidase, collagenase, and elastase. (A) Hyaluronidase enzyme inhibition was compared with tannic acid as a positive control. (B and C) Collagenase and elastase inhibitions were compared with standard EGCG as a positive control. * p < 0.05 and ** p < 0.01 indicate significance levels compared with all treatments.

The anti-aging potential of active compounds can be explored through various mechanisms. The 1:5 of Cur: EGCG combination was selected for its ability to enhance SIRT1 protein expression and activity, a key anti-aging protein in the cell nucleus. Another key process in anti-aging involves inhibiting the degradation of ECM. Hyaluronic acid, collagen, and elastin, secreted by the skin cells to form the skin's supportive tissue structure, are susceptible to breakdown by enzymes such as hyaluronidase, collagenase, and elastase. The degradation of these components is a major factor in the aging process (Frantz et al., 2010; Lim et al., 2020). The results are presented as %inhibition of hyaluronidase, collagenase, and elastase. The inhibitory effects of 1:5 of Cur: EGCG are shown in Fig. 6. Cur, EGCG, and the 1:5 Cur: EGCG combination exhibited inhibitory effects on hyaluronidase in the range of 13–20 % at the same concentration as tannic acid (85.91 ± 1.30 %) that used as a positive control, as shown in (Fig. 6A). Cur and 1:5 Cur: EGCG exhibited inhibitory effects on collagenase enzyme in the range of 35–44 % at the same concentration as EGCG and standard EGCG (approximately 80 %) that used as a positive control, as shown in (Fig. 6B). Among the samples tested against the elastase (Fig. 6C), 1:5 of Cur: EGCG (51.76 ± 2.30 %) exhibited a higher inhibitory effect than that from Cur (16.32 ± 0.03 %), and EGCG alone (23.09 ± 0.69 %) (*p* < 0.05). EGCG can inhibit hyaluronidase, collagenase, and elastase, so EGCG is commonly used as a positive control for collagenase and elastase enzymes inhibition methods (Avadhani et al., 2017). The combination has the highest inhibitory effect on the elastase enzyme. Inhibiting the elastase was a synergistic effect between Cur and EGCG at a ratio of 1:5. The elastase enzyme breaks down elastin, a flexible fiber of the skin dermis (Thring et al., 2009).

### 3.7 Stability, release study, and skin penetration of the combination-loaded NLCs in emulgel

#### 3.7.1 Stability of the combination-loaded NLCs in emulgel

**Fig. 7:**
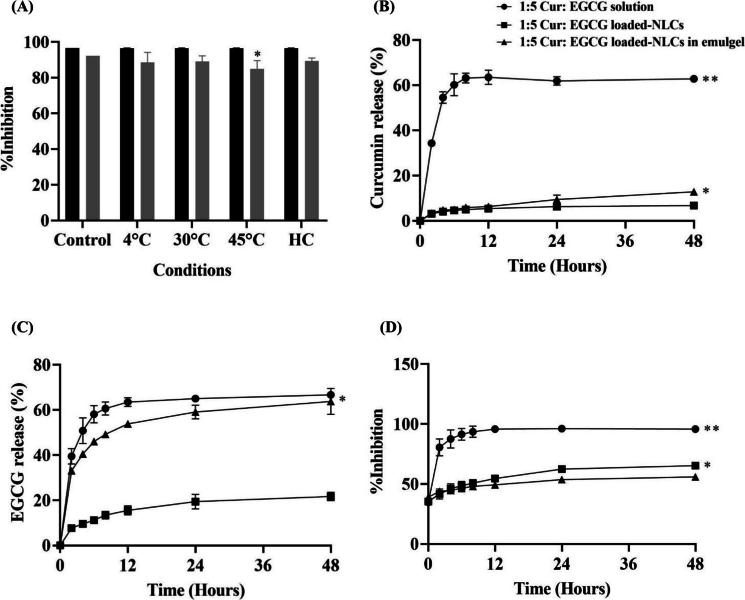
Stability study and skin release study from the combination. (A) Antioxidant activity from DPPH assay of 1:5 of Cur: EGCG loaded NLCs in emulgel at before and after stability test. * *p* < 0.05 compared between before and after stability test (*n* = 3). Release profile of (B) Cur, (C) EGCG, and (D) % inhibition from DPPH assay from 1:5 of Cur: EGCG solutions, 1:5 of Cur: EGCG loaded-NLCs, and 1:5 of Cur: EGCG loaded-NLCs in emulgel. * p < 0.05 and ** *p* < 0.01 indicate significant levels compared with all samples (n = 3).

After preparing, the NLCs and emulgel were mixed in a 1:1 ratio with moderate stirring to prepare the formulation. The final formulation filled in an aluminum tube was evaluated for stability under various conditions, including heating-cooling for 6 cycles, 30 °C, 4 °C and 45 °C for 28 days. Physicochemical characteristics such as color, pH (4.71 ± 0.07) and viscosity did not statistically change (30–37 Pas), except for the color change from pale yellow to yellow at HC, and 45 °C (data not shown). The formulations in various conditions were analyzed using the antioxidant activity by the DPPH method, as shown in Fig. 7A. The formulation before and after the stability test was stable with antioxidant activity at approximately 85 %. Whereas at 45 °C, antioxidant activity significantly decreased after 28 days (84.93 ± 4.66 %). The instability of the combination-loaded NLCs in emulgel at an elevated temperature was attributed to the melting of the lipid structures, leading to NLC decomposition and subsequent degradation of the active ingredients. This degradation resulted in a reduced percentage of inhibition in antioxidant activity tests compared to day 0 (Mahant et al., 2020).

#### 3.7.2 Release study and skin penetration study

The release profiles of 1:5 of Cur: EGCG solutions, 1:5 of Cur: EGCG-loaded NLCs, and 1:5 of Cur: EGCG-loaded NLCs in emulgel are illustrated in Fig. 7. After 48 h, the release of Cur into the 20 % MeOH in PBS, pH 5.5 were quantified as follows: 62.84 ± 1.13 % from the 1:5 of Cur: EGCG solution, 6.78 ± 0.72 % from the 1:5 of Cur: EGCG-loaded NLCs, and 12.82 ± 0.99 % from the 1:5 of Cur: EGCG-loaded NLCs emulgel. The combination of 1:5 Cur: EGCG-loaded NLCs in emulgel, unlike Cur, showed a higher release for EGCG. After 48 h, the release of 1:5 of Cur: EGCG solutions, 1:5 of Cur: EGCG-loaded NLCs, and 1:5 of Cur: EGCG-loaded NLCs were as follows: 66.67 ± 0.07, 21.71 ± 1.90 and 63.77 ± 5.76 %, respectively (Fig. 7C). From the Fig. 7C., EGCG was readily released from the solutions and the NLCs in emulgel significantly higher than that from the NLCs (*p* < 0.05). These results were due to the greater hydrophilicity of EGCG than the Cur. In the lipid environment of NLCs, the release of both actives was retarded; however, the NLCs incorporated into the emulgel exhibited a greater release of the more hydrophilic EGCG. The selection of the base formulation is important for the release behavior. The antioxidation activity was also monitored using the DPPH assay as shown in Fig. 7D. The free radical scavenging activity expressed as percent inhibition of 1:5 of Cur: EGCG solution, 1:5 of Cur: EGCG-loaded NLCs, and 1:5 of Cur: EGCG-loaded NLCs in emulgel at 48 h were 95.78 ± 1.77, 65.37 ± 0.51, and 55.99 ± 2.09 %, respectively. From the Fig. 7D., the 1:5 of Cur: EGCG loaded in NLC and emulgel exhibited a gradual increase in antioxidant activity. Cur and EGCG were controlled release gradually over time, consistent with the properties of NLCs. EGCG could release more than Cur because the structure of EGCG is more hydrophilic than Cur (Elmowafy and Al-Sanea, 2021). EGCG was also located in the core of NLCs near the surfactant to release quickly (Nagle et al., 2006).

The comparative penetration of Cur and EGCG from five formulations: blank NLCs, emulgel, 1:5 of Cur: EGCG in emulgel, 1:5 of Cur: EGCG-loaded NLCs, and 1:5 of Cur: EGCG-loaded NLCs in emulgel were observed. The Franz diffusion method evaluated NLCs in emulgel through the artificial skin membrane (Strat-M®). The active compounds of the combination were not found in the receptor chambers at any time points, indicating limited permeation across the membrane. After 24 h of sample application on the artificial skin model, followed by the removal of excess product, cumulative accumulation of the Cur: EGCG (1:5) from the formulation was observed in the Strat-M® membrane. The percentages of the penetrated amount of each compound (% of initial) are shown in Table 3. The penetration of Cur from 1:5 of Cur: EGCG-loaded NLCs in emulgel (23.88 ± 1.71 %) was significantly higher (*p* < 0.05) when compared with the 1:5 of Cur: EGCG-loaded NLCs (12.82 ± 4.11 %) and 1:5 of Cur: EGCG in emulgel (9.97 ± 5.42 %). Cur from 1:5 of Cur: EGCG-loaded NLCs in emulgel remained in the membrane approximately two-fold higher than that from the 1:5 of Cur: EGCG-loaded NLCs and 1:5 of Cur: EGCG in emulgel. Similar to the Cur, EGCG from 1:5 of Cur: EGCG-loaded NLCs in emulgel showed higher membrane deposition (22.79 ± 4.65 %), approximately two-fold higher than the combination-loaded NLCs (10.49 ± 1.78 %) and ten-fold higher than the combination in emulgel (2.99 ± 0.88 %). Combination-loaded NLCs in emulgel showed greater penetration into the skin because of the emulgel formulation contained both gel and various liquid lipids in the lipid phase, enabling the solubilization and partition into the skin (Calderon-Jacinto et al., 2022; Kesharwani et al., 2020; Lane, 2013). Because the size of NLCs is approximately 141.2 nm—well below 300 nm—they can penetrate the skin (Shakoor et al., 2023), allowing unreleased Cur to serve as a reservoir for subsequent release. The formulation-containing gel can help increase the hydration of the skin barrier, resulting in the skin being highly moisturized and allowing active ingredients to pass through the skin (Kumar et al., 2016). One of the most important release mechanisms of SLNs and NLCs is their occlusive effect, which results from forming a film layer on the skin. When the lipid nanodispersion is applied to the skin, an adhesive occlusive film layer and skin hydration occur. Furthermore, the presence of surfactant in the system, disruption of the intercellular lipid structure, and/or interaction with the intracellular proteins of the horny support the nanoparticle skin penetration (Patel et al., 2019). In addition, lipid composition influences the NLC interaction with the skin membrane. After the penetration into the skin barrier, drug diffusion and distribution along with lipid matrix erosion can occur (Chen et al., 2012; Wani et al., 2020). For deeper skin penetration, intact particles entrapped with a fluorescent dye of sizes <600 nm can accumulate in the hair follicles, acting as a reservoir for sustained release (Correia et al., 2023).

**Table 3:** Percentage of active compounds in Strat-M® membrane analyzed using HPLC. Results are shown as the means ± S.D. (n = 3). * indicates significance level at *p* < 0.05.

Sample	Active compounds in Strat-M® membrane (%)	
	Cur	EGCG
Blank NLCs	–	–
Emulgel base	–	–
1:5 Cur: EGCG in emulgel	9.97 ± 5.42	2.99 ± 0.88
1:5 Cur: EGCG-loaded NLCs	12.82 ± 4.11	10.49 ± 1.78*
1:5 Cur: EGCG-loaded NLCs in emulgel	23.88 ± 1.71*	22.79 ± 4.65**

## 4 Conclusion

The 1:5 combination of Cur and EGCG exhibited superior total phenolic content, potent antioxidant activity, and enhanced SIRT1 protein expression, while remaining non-toxic to immortalized human keratinocyte HaCaT cells. Furthermore, the NLC-loaded combination exhibited particle sizes ranging from 100 to 200 nm with a PDI below 0.3, maintaining stability at 4 °C. This formulation demonstrated superior inhibition of elastase and collagenase, key enzymes involved in skin aging. When incorporated into an emulgel, the NLCs showcased high release efficiency and effective skin penetration. Collectively, these findings suggest that the 1:5 Cur: EGCG combination holds promise as a potent cosmetic ingredient with remarkable anti-aging properties, including wrinkle prevention and radical scavenging activity.

## Funding

This research project was supported by Fundamental Fund 2024, 10.13039/501100002842Chiang Mai University (FF002/2567) and also Thailand Science Research and Innovation (TSRI) [Grant numbers FRB670083/0162].

## CRediT authorship contribution statement

**Chidchanok Prathumwon:** Writing – original draft, Validation, Methodology, Investigation, Formal analysis, Data curation, Conceptualization. **Songyot Anuchapreeda:** Writing – review & editing, Supervision, Resources, Methodology, Investigation. **Kanokwan Kiattisin:** Writing – review & editing, Validation, Resources, Methodology. **Pawaret Panyajai:** Methodology, Investigation, Formal analysis. **Panikchar Wichayapreechar:** Methodology, Investigation, Data curation. **Young-Joon Surh:** Writing – review & editing, Supervision. **Chadarat Ampasavate:** Writing – review & editing, Visualization, Supervision, Resources, Investigation, Conceptualization.

## Declaration of competing interest

The authors declare that they have no conflicts of interest to disclose and the research did not receive any specific grant from funding agencies in the public, commercial, or not-for-profit sectors.

## Data Availability

Data will be made available on request.
