# Supplementary material for: Curcumin and EGCG combined formulation in nanostructured lipid carriers for anti-aging applications
Source: Int J Pharm X. 2025 Feb 25;9:100323. doi: 10.1016/j.ijpx.2025.100323 (PMC11923819; doi:10.1016/j.ijpx.2025.100323)
Supplement: Supplementary file 1 — Supplementary material [file mmc1.docx]

**Supplementary file**

Curcumin and EGCG Combined Formulation in Nanostructured Lipid Carriers for Anti-Aging Applications

Chidchanok Prathumwon ^a^, Songyot Anuchapreeda ^b^, Kanokwan Kiattisin ^a^, Pawaret Panyajai ^b^, Panikchar Wichayapreechar ^d^, Young-Joon Surh ^e^, Chadarat Ampasavate ^a,c,*^

^a^ Department of Pharmaceutical Sciences, Faculty of Pharmacy, Chiang Mai University,

Chiang Mai 50200, Thailand

^b^ Division of Clinical Microscopy, Department of Medical Technology, Faculty of Associated Medical Sciences, Chiang Mai University, Chiang Mai, 50200, Thailand

^c^ Center for Excellence in Pharmaceutical Nanotechnology, Faculty of Pharmacy, Chiang Mai University, Chiang Mai 50200, Thailand

^d^ Department of Cosmetic Science, School of Pharmaceutical Sciences, University of Phayao, Phayao 56000, Thailand

^e^ College of Pharmacy, Seoul National University, Seoul 151-741, South Korea

Corresponding author: Chadarat Ampasavate*

Mailing address: Department of Pharmaceutical Sciences, Faculty of Pharmacy, Chiang Mai

University, Chiang Mai 50200, Thailand

Tel.: +66 53 944 342; Fax: +66 053 944 390

Mobile phone: +66 994 297 451

E-mail address: chadarat.a@cmu.ac.th

**Table S1.** Optimized gradient elution for simultaneous estimation of Cur, Res, and EGCG.

| Time | Mobile phase | | |
| --- | --- | --- | --- |
|  | 0.1% Phosphoric acid in water (%) | Acetonitrile (%) | Methanol (%) |
| 0 | 83.5 | 8.25 | 8.25 |
| 7 | 83.5 | 8.25 | 8.25 |
| 12 | 40 | 55 | 5 |
| 27 | 40 | 55 | 5 |
| 28 | 83.5 | 8.25 | 8.25 |
| 35 | 83.5 | 8.25 | 8.25 |

**Table S2.** The combinations of active compounds in molar ratios.

| Sample | Active compounds (M) | | |
| --- | --- | --- | --- |
|  | Cur | Res | EGCG |
| 1 | 1 | - | - |
| 2 | - | 1 | - |
| 3 | - | - | 1 |
| 5 | 1 | 1 | - |
| 6 | 1 | 5 | - |
| 7 | 1 | 10 | - |
| 8 | 1 | - | 1 |
| 9 | 1 | - | 5 |
| 10 | 1 | - | 10 |
| 11 | 1 | 1 | 1 |
| 12 | 1 | 5 | 1 |
| 13 | 1 | 1 | 5 |
| 14 | 1 | 5 | 5 |

**Table S3.** Ingredients of NLCs preparation.

| Ingredient | Formulation (% w/w) | | | | | | |
| --- | --- | --- | --- | --- | --- | --- | --- |
|  | F1 | F2 | F3 | F4 | F5 | F6 |  |
| Glyceryl behenate | 1.75 | 1.75 | 1.75 | 1.75 | 1.75 | 1.75 |  |
| Cetearyl alcohol | 1.75 | 1.75 | 1.75 | - | - | - |  |
| Cocoa butter | - | - | - | 1.75 | 1.75 | 1.75 |  |
| Krill oil | 1.5 | - | - | 1.5 | - | - |  |
| Meadowfoam seed oil | - | 1.5 | - | - | 1.5 | - |  |
| Sweet almond oil | - | - | 1.5 | - | - | 1.5 |  |
| Sorbitan oleate/Polysorbate 80 | 10 | 10 | 10 | 10 | 10 | 10 |  |
| Distilled water q.s. | 100 | 100 | 100 | 100 | 100 | 100 |  |

**Table S4.** Solubility of Cur and EGCG in krill oil, meadowfoam seed oil, and sweet almond oil.

| Liquid lipid | Solubility (mg/mL) | |
| --- | --- | --- |
|  | Cur | EGCG |
| Krill oil | 0.20 | 0.22 |
| Meadowfoam seed oil | 0.33 | 0.40 |
| Sweet almond oil | 0.33 | 0.50 |

**Fig. S1.** The product images of empty NLCs, 1:5 Cur: EGCG loaded-NLCs, emulgel, 1:5 Cur: EGCG loaded-NLCs in emulgel.


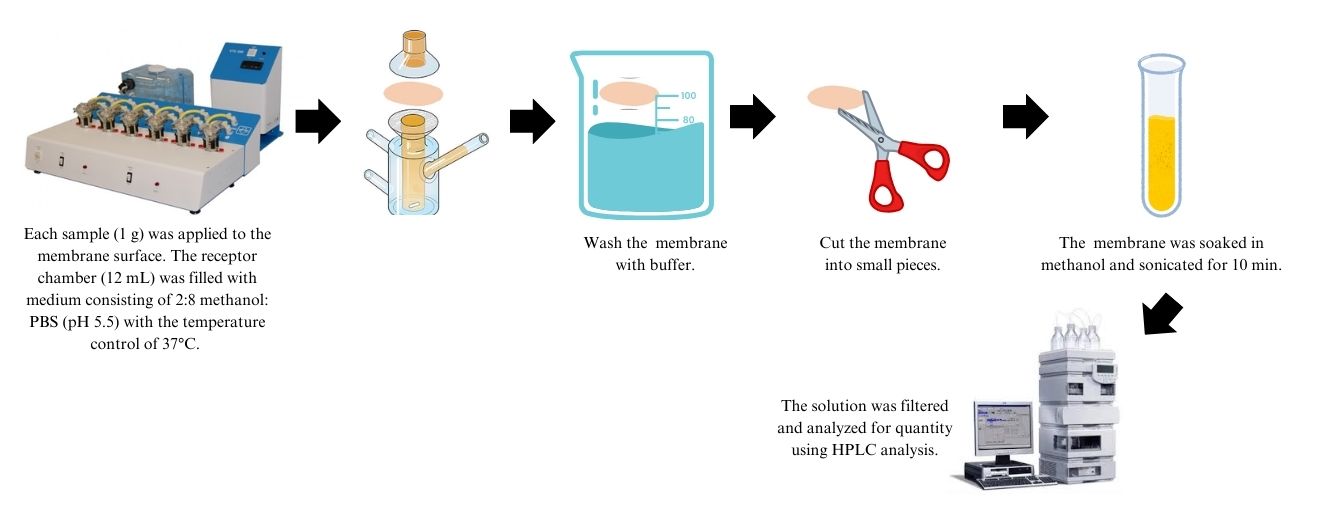
**Fig. S2.** A schematic illustration for the skin penetration study.
